# Single-breath-hold whole-heart coronary MRA in healthy volunteers at 3.0-T MRI

**DOI:** 10.1186/2193-1801-3-667

**Published:** 2014-11-11

**Authors:** Yuji Iyama, Takeshi Nakaura, Masafumi Kidoh, Tetsuya Kawahara, Naritsugu Sakaino, Kazunori Harada, Tomoyuki Okuaki, Yasuyuki Yamashita

**Affiliations:** Diagnostic Radiology, Amakusa Medical Center, Kameba 854-1, Amakusa, Kumamoto, 863-0046 Japan; Department of Diagnostic Radiology, Graduate School of Medical Sciences, Kumamoto University, Honjo 1-1-1, Kumamoto, Kumamoto, 860-8556 Japan; Department of Cardiovascular Internal Medicine, Amakusa Medical Center, Kameba 854-1, Amakusa, Kumamoto, 863-0046 Japan; Department of Surgery, Amakusa Medical Center, Kameba 854-1, Amakusa, Kumamoto, 863-0046 Japan; MR Clinical Scientist Philips Healthcare AsiaPacific, 13-37 Kohnan 2-chome Minato-ku, Tokyo, 108-8507 Japan; Department of Diagnostic Radiology, Graduate School of Medical Sciences, Kumamoto University, Honjo 1-1-1, Kumamoto, Kumamoto, 860-8556 Japan

**Keywords:** Single-breath-hold whole-heart MRA, Imaging, 3-T MRI

## Abstract

**Background:**

The purpose of this study was to investigate the feasibility of single-breath-hold whole-heart MRA with a 3-T system. Ten healthy male volunteers underwent single-breath-hold whole-heart coronary MRA at 3 T. We assessed acquisition time, scores of image quality of coronary artery (RCA: proximal, middle and distal, LAD: main, proximal, middle and distal, LCX: proximal and distal) and the visualized vessel length of RCA, LAD and LCX.

**Findings:**

Mean acquisition time was 37.7 ± 5.2 sec. Coronary branch was successfully depicted in 67/80 branches (84%) in the 10 healthy volunteers with diagnostic image quality. And, the average visible RCA, LAD and LCX vessel length were 83.4 ± 22 mm and 59.6 ± 24 mm.

**Conclusions:**

3-T MRI with single-breath-hold 3D whole-heart coronary MRA can yield adequate image quality. Further study is needed to evaluate the clinical benefit of this technique.

## Introduction

Whole-heart coronary MR angiography (MRA) is generally carried out during free breathing with a respiratory gating method using navigator echo techniques, which track the motion of the right hemi-diaphragmatic dome. The advantage of free breathing technic is unnecessary to stop breathing. However, the major drawback of this free breathing technique is the relatively long acquisition time, ranging from 10 to 20 minutes (Sakuma et al. 2005). The technic of single-breath hold technic could shorten the total scan time because it can reduce influence of respiratory motion. There were some reports about whole-heart coronary MRA with 1.5 T MRI during single breath hold (Makowski et al. 2012; Okada et al. 2011; Nassenstein et al. 2008). In general, increased SNR by 3-T MRI enables us to increase image quality and exam speed compared to 1.5-T MRI; however, high-field systems still pose challenges in terms of their specific absorption rate (SAR) and radiofrequency (RF) excitation uniformity, especially in cardiac MRI (Nezafat et al. 2006).

Recently introduced 3-T systems encompass two major innovative technologies for coronary MRA. The Direct Digital RF receiver technology digitizes the MR signal at the patient, and the fiber-optic connection from the coil to the image reconstructor enables lossless broadband data transmission. Previous reports suggested that this technique improved the dynamic range of the RF receiver and resulted in an improved signal-to-noise ratio (SNR) (Ruipeng et al. 2009). Dual-source radiofrequency transmission with patient-adaptive local radiofrequency shimming enables uniform RF shimming for cardiac MRI (Mueller et al. 2012). This technique reduces dielectric shading, improves B 1 homogeneity, and increases image contrast by T2 preparation prepulse (T2prep) with high-power refocusing pulses. These new technologies in 3-T MRI might increase the SNR at cardiac MRI, and enable us to shorten the acquisition time of whole-heart coronary MRA with adequate image quality. However, to our knowledge, there is no published protocol for single-breath-hold whole-heart MRA with recent 3-T systems.

The purpose of this study was to investigate the feasibility of single-breath-hold whole-heart MRA with a 3-T system on healthy volunteers.

## Methods

### Subjects

This prospective study received Amakusa medical center institutional review board approval, and prior informed consent to participate was obtained from 10 healthy male volunteers. All volunteers were imaged consecutively between August 2012 and September 2012. Ages ranged from 24 to 53 (mean 35.6 ± 11.0) years old, and heart rate ranged from 45 to 75 (mean 59.8 ± 9.6) beats per minute. Table 1 shows the characteristics of the volunteers.

**Table 1:** **Volunteer’s objective data**

Number	Sex	Age (years)	HR (beats/min)	Total scan time (sec)	Height (cm)	Weight (kg)	LAD	LCX	RCA
1	Male	47	60	45	172	67	98.9	53.7	131.3
2	Male	53	75	45	171	76	116.8	77.6	118
3	Male	46	60	40	168	72	75.8	20.3	130.2
4	Male	24	75	38	167	63	49.1	76.9	77.8
5	Male	31	55	34	174	58	75.4	37.9	140.9
6	Male	28	50	31	175	65	97.9	74.5	97.9
7	Male	45	55	32	168	76	95.2	86.1	80.4
8	Male	24	45	40	166	61	115	55.2	148
9	Male	25	60	40	175	63	90.6	83	76.3
10	Male	33	63	32	170	63	57.8	49.4	148
average		35.6 ± 11.0	59.8 ± 9.6	37.7 ± 5.2	170.6 ± 3.3	66.4 ± 6.3	87.3 ± 22.5	61.5 ± 21.7	114.9 ± 29.3

Note: Data are shown as the mean ± standard deviation.

### MR angiography acquisition

All ten subjects were imaged by 3-T MRI (Ingenia, Philips Medical Systems) using a 16-element phased-array Direct Digital RF receiver coil and vector electrocardiographic (VCG) gating (Fischer et al. 1999). A multi-slice gradient echo (TR = 2.6 ms; TE = 1.27 ms; α = 20°) scout scan was acquired in 3 orthogonal orientations for localization of the volume for whole-heart imaging. After the 3D scout scan, an axial ECG-triggered, segmented steady-state free precession (SSFP) cine image series (TR = 2.6 ms, TE = 1.28 ms, α = 45°, and temporal resolution of 10 ms) at the level of the proximal-to-mid right coronary artery (RCA) was also obtained during a single breath hold. This was carried out for visual determination of the most quiescent period in the cardiac cycle, which was subsequently used to set the trigger delay and the shot duration. In addition, an ECG-triggered segmented 3 D SSFP sequence using the proposed undersampling scheme was implemented and in-vivo measurements were performed in expiration.

Subsequently, 3D whole-heart turbo field echo (TFE) coronary MRA was acquired using this visually identified trigger delay and shot duration. We visually measured the rest period of the RCA during diastole phase, and defined the shot duration as long as possible. No intravenous contrast agents were used; and, a T2prep (TE = 70 ms) was used to increase the contrast of natural T2 differences between blood and myocardium. The T2prep technic could yield four times more refocusing pulses than that of without T2 prep technic for turbo gradient echo coronary MRA in 3-T MRI. Spectrally selective fat saturation was also utilized for additional endogenous image contrast enhancement between the coronary blood pool and the surrounding fat. The detailed scanning parameters are shown in Table 2.

**Table 2:** **Scan parameters**

FOV	320 mm
RFOV	80%
ACQ voxel size	2.0 × 2.0 × 2.0 mm
Reconstructed voxel size	1.0 × 1.0 × 1.0 mm
Slices	124
Slice thickness	2 mm (1 mm reconstruction)
Scan mode	3D
Scan technique	FFE
Fast imaging mode	TFE
Shot mode	Multishot
Profile order	Low-high
Turbo direction	Radial
TR/TE	3.4/1.5 msec
Fat suppression	SPIR
NSA	1
Half-scan	Y factor: 0.625, Z factor: 0.9
Flip angle	12 deg.
T2prep	TE: 70 ms, 4 RF pulses
SENSE factor	Phase direction 2, Slice direction 1.5

### Image analysis

To evaluate the image quality, we performed qualitative image analysis of axial images and curved MPR images on a PACS viewer (Synapse, Fuji Film Medicals). Two board-certified radiologists with 8 and 5 years of experience with cardiac MRI independently graded overall image quality according to a segmentation scheme recommended by the American College of Cardiology and the American Heart Association (ACC/AHA) (Scanlon et al. 1999). The RCA was subdivided into three segments (proximal, middle and distal), the LAD into four segments (main, proximal, and middle) and the LCX into two segments (proximal and distal). We defined these segment as follows; (#1 in AHA: RCA proximal segment, #2 in AHA: RCA middle segment, #3 in AHA: RCA distal segment, #5 in AHA: LAD main segment, #6 in AHA: LAD proximal segment, #7 in AHA: LAD middle segment, #11 in AHA: LCX proximal segment and #13 in AHA: LCX distal segment). We used a 5-point subjective scale for qualitative image analysis: 4, excellent (the vessel was well depicted with sharply defined borders); 3, good (the vessel was adequately visualized, with confidence in the diagnosis, only mildly blurred borders); 2, fair (coronary vessel was visible, but confidence in the diagnosis was low due to moderately blurred borders); 1, poor (coronary vessel was barely seen or was obscured by noise); and 0, not visualized (Sakuma et al. 2005; Wu et al. 2007). The visualized vessel length of RCA, LAD and LCX were also measured. Interobserver disagreements were resolved by consensus.

## Results

Single-breath-hold whole-heart coronary MRA was technically successful in all 10 volunteers. Total scan times varied from 31 sec to 45 sec because we changed the shot duration for each volunteer.

**Figure 1:**
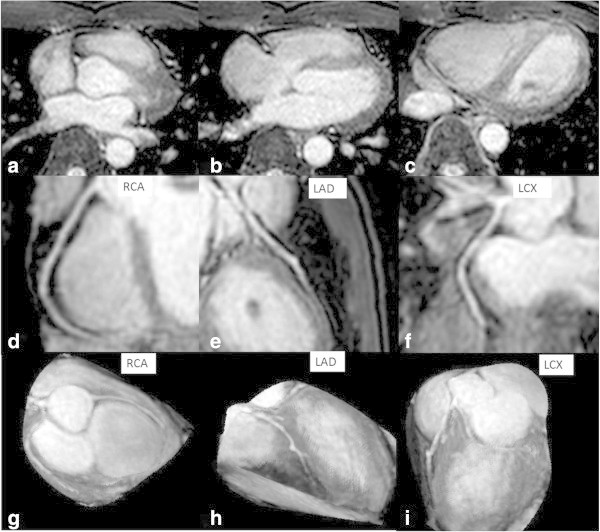
**A 24-year-old volunteer was imaged by single-breath-hold whole-heart MRA with a 3-T system.** His heart rate was 51 beats per minute, shot duration time 150 ms and scan time 31 sec (volunteer number 8 in Table 1). We showed his original image **(a, b, and c)**, curved MPR (multi planner reconstruction) image **(d, e, and f)** and PWMIP (partial width maximum intensity projection) image **(g, h, and i)**. All coronary branches were successfully depicted with diagnostic image quality. The qualitative image score is (RCA proximal segment: 4, RCA middle segment: 4, RCA distal segment: 4, LAD main segment: 4, LAD proximal segment: 3, LAD middle segment: 3, LCX proximal segment: 4, LCX distal segment: 4).

The TFE sequence provided uniform, depictions of coronary arteries, as shown in Table 3. Table 3 show the results of the qualitative analysis. Coronary branch was successfully depicted in 62/80 branches (77.5%) by reader 1, 66/80 branches (82.5%) by reader 2 and 67/80 branches (83.8%) by consensus in the 10 healthy volunteers with diagnostic image quality (score: 4 or 3). The average visible RCA, LAD and LCX vessel length were 114.9 ± 29.3 mm, 83.4 ± 22 mm and 59.6 ± 24 mm. Figure 1 shows an image (original image, curved MPR and PWMIP) of a volunteer, for whom three coronary branches were successfully depicted with diagnostic image quality.

**Table 3:** **Image analysis**

	Reader 1	Reader 2	Consensus
Well-depicted coronary branch (score: 4 or 3)	62/80 (77.5%)	66/80 (82.5%)	67/80 (83.8%)
RCA proximal segment (#1)	3.5 ± 0.7	3.7 ± 0.7	3.6 ± 0.7
RCA middle segment (#2)	3.5 ± 0.7	3.7 ± 0.7	3.6 ± 0.7
RCA distal segment (#3)	2.5 ± 0.7	3.7 ± 0.7	3.1 ± 1.0
LAD main segment (#5)	2.7 ± 0.5	3.6 ± 0.7	3.3 ± 0.7
LAD proximal segment (#6)	2.4 ± 0.5	3.5 ± 0.7	3.1 ± 0.7
LAD middle segment (#7)	2.7 ± 0.5	3.3 ± 0.9	3.0 ± 0.7
LCX proximal segment (#11)	2.8 ± 0.6	3.2 ± 0.9	3.2 ± 0.8
LCX distal segment (#13)	2.7 ± 0.5	3.3 ± 0.9	3.1 ± 0.7

Note: Data are shown as the mean ± standard deviation.

## Findings

The purpose of this study was to investigate the feasibility of single-breath-hold whole-heart MRA with a 3-T system. We assessed acquisition time, scores of image quality of coronary artery of ten volunteers. Mean acquisition time was 37.7 ± 5.2 sec. The average visible RCA, LAD and LCX vessel length were 83.4 ± 22 mm and 59.6 ± 24 mm.

## Discussion

To our knowledge, this is the first report on the clinical feasibility of single-breath-hold 3D whole-heart coronary MRA in 3-T MRI.

Although single-breath-hold 3D whole-heart coronary MRA has been performed at 1.5-T MRI using the SSFP technique, the increased B1 field inhomogeneity and SAR limit the consistency of SSFP in coronary images in 3.0-T MRI (Nezafat et al. 2006; Stuber et al. 2002). There are a few reports about 1.5 T single-breath-hold coronary MRA (Makowski et al. 2012; Lim et al. 2013). Because, this technic has disadvantage of breathing hold for a long time, therefore, patients with a respiratory disease have difficulty in receiving examination. However, 3 T MRI can shorten scan time, and patients may not need a long breath-hold. Therefore, TFE sequence that has better tolerance to field inhomogeneity than SSFP has been used for coronary MRA at 3.0 T. However, a major drawback of coronary MRA with TFE is that the SNR of the coronary arteries and the blood-myocardial contrast are not as high as those of SSFP sequence (Maintz et al. 2004). And, there were few reports about single-breath-hold 3D whole-heart coronary MRA with our study suggested that a recent 3-T MRI might offer adequate SNR in spite of having the short acquisition time of whole-heart coronary MRA. We did not know the reason the result of our study was better than that of previous report about single-breath-hold 3D whole-heart coronary MRA (Nezafat et al. 2006; Stuber et al. 2002). There are many factors to improve image quality ( for example, recent 3-T MRI system, such as increasing the number of channel coils, the Direct Digital RF receiver technology and the dual-source radiofrequency transmission). We think that the increased refocusing pulses (T2prep) mainly overcome the decreased signal with the TFE sequence in free breathing coronary MRI. In general, the image contrast upon using TFE MRI is dependent on the number of prepulses. Botnar et al. state that the combined approach of free-breathing navigator-gated and slice-tracked 3D coronary MRA together with a T2prep and a shorter acquisition window resulted in an improved CNR between coronary blood and myocardium and thereby allowed for better definition of the coronary vessels (Maintz et al. 2004). And, T2 prep technic may be useful in breath-hold coronary MRI.

Actually, our study shows that the average visible RCA vessel length of 114.9 ± 29.3 mm compared favorably with earlier reported navigator-gated bSSFP (80 ± 40 mm) and gradient echo sequences (95 ± 22 mm) in RCA at 3.0 T (Kaul et al. 2004). Therefore, a large value of T2prep could improve image quality. Sahar et al. reported that T2 prep technic increased image quality compared to non T2 prep technic (Soleimanifard et al. 2013). Mueller et al. state that dual-source RF transmission with RF shimming results in an optimized SAR distribution, thereby reducing local SAR peaks (Mueller et al. 2012). As such, we can decrease SAR by using multitransmit technology.

Our study had a number of limitations. First, we only evaluated 10 healthy volunteers. Future studies are needed to evaluate more patients with suspected coronary heart disease. Second, our evaluation only involved a protocol for single-breath-hold whole-heart MRA with the recent 3-T systems. Future studies are needed to compare the protocol for a single breath hold and the protocol for free breathing with a respiratory gating method. Third, total scan time in our study is comparatively long (from 31 sec to 45 sec). However, increasing reduction factor in parallel imaging be able to shorten total scan time in future studies. Forth, in our study, the image quality of the distal segments of the LCX and LAD were significantly poorer than the image quality of the RCA. When the distal segments were excluded, the image quality between the coronary arteries was not significant different. We believe that the poor quality of the LCX and LAD was caused by the small diameter of its distal segment. The result of the previous report was the same kind (Kim et al. 2006).

In conclusion, 3-T MRI with single-breath-hold 3D whole-heart coronary MRA can yield adequate image quality. Further study is needed to evaluate the clinical benefit of this technique.
